# The League of Mercy: Annual Meeting at St. James's Palace

**Published:** 1915-12-25

**Authors:** 


### THE LEAGUE OF MERCY.

### Annual Meeting at St. James's Palace.

THE sixteenth annual meeting of Presidents of the League of Mercy was held at St. James's Palace on Tuesday, December 21, under the presidency of Lord Farquhar, G.C.V.O. H.R.H. Princess Alexander of Teck, the Lady Grand President, was present.

### Letter from the King.

The CHAIRMAN read the following gracious letter which had been sent by Lord Stamfordham by command of their Majesties the King and Queen:

"The King and Queen are gratified to learn that, despite the many adverse circumstances and the rival claims arising out of the war, the collections by the League of Mercy again show some advance upon those of the previous year. The fact that during the seventeen years of its existence the League has distributed no less than a quarter of a million sterling among the hospitals in and around London is an achievement of which all the officers and workers of that organisation must be justly proud. Their Majesties heartily congratulate the Presidents, Vice-Presidents, and members of the League on the substantial result of their sustained and arduous labours.—Yours very truly, STAMFORDHAM."

He was sure all present would be greatly encouraged by those gracious words.

### Lord Farquhar was sorry to say that the League had again to regret the absence of the Grand President, Prince Alexander of Teck, who was actively engaged in serving his country in France. He was sure they could congratulate themselves on the presence of her Royal Highness Princess Alexander of Teck, who would present the decorations to whom they were due. Her Royal Highness had always taken a most interested and active part in the work of the League of Mercy, as indeed had several members of the Royal Family, notably her Royal mother, the Duchess of Albany, and Princess Victoria of Schleswig-Holstein. This year, notwithstanding the extraordinary way in which the British public had responded to many appeals, especially for our wounded sailors and soldiers, he was pleased to state that the collections showed an increase over those of last year, so that the same sum could be given to King Edward's Fund and to the various hospitals. When the collections were started at the beginning of this year, it was feared that they would not be so successful as heretofore, consequently they were very anxious to know how matters were progressing, and two separate schemes for making up any deficit were considered. One of these was the exhibition of a special film for the League of Mercy, which was to be shown at all the picture-palaces in London. This scheme, which was originated by Miss Olive Andrews, interested him very much, and he wished to congratulate her on her success. The other plan was a house-to-house envelope collection, and that scheme also, he was happy to say, had been very successful. Outside these special efforts all that was possible had been done to collect funds in the usual way, and the results had been very satisfactory. There had been several changes since he had the honour of meeting the workers for the League last year. The first and most important—upon which he congratulated all the members of the League—was that their old friend and member of the Council, Sir Frederick Green, had kindly consented to take the position of Honorary Treasurer to the League. He was sure that under the circumstances the League could not have had a better man to perform this duty, and he would receive their gratitude for it. He had also to report that Brigadier-General Kempster regarded it as his duty to resign his position as Secretary in order that he might re-enter the Army. They were very sorry to part with him, and regretted his absence from the meeting. He had been succeeded by Mr. Arthur Franklyn, who was lately Secretary to the Royal Waterloo Hospital for Women and Children. Mr. Franklyn had already helped them a great deal, and he had ably developed and worked the envelope system. There were two gentlemen coming up to-day for election to the Council whom the Council would heartily welcome—namely, Lord Hill and Colonel the Right Hon. Mark Lockwood. His Lordship, in conclusion, congratulated the Vice-Presidents and members of the League on the very successful way in which they had worked during the past most trying year, the result of which was an increase, instead of a decrease, in the amounts collected. But still he felt it to be his duty to urge them, with all the power he possessed, to continue their energy and work, and, if possible, increase it, so that all the contributions could be continued with all the good which followed in its train.

#### SOME BUSINESS ITEMS.

The Right Hon. the Lord Mayor of London (Sir Charles Cheers Wakefield) proposed: "That the Right Hon. the Lord Farquhar, G.C.V.O., be appointed Chairman; Sir Frederick Green, Deputy-Chairman; and Captain A. E. Speer, Hon. Secretary, respectively." It was evident to him that this work must have gripped the hearts and imaginations of the gentlemen named, otherwise the League would not have the privilege of counting them as officers. Mr. Montagu Sharpe seconded, remarking that as a member of the Council for ten years he could speak of the ability and zeal with which the gentlemen now proposed carried on their work. The resolution, on being put by the Lord Mayor, was carried unanimously.

On the motion of Mr. Philip Walker, seconded by Mr. H. W. Keay, the Executive Committee were re-appointed, with Viscount Hill and Colonel the Right Hon. Mark Lockwood, C.V.O., M.P., as new members.

Mr. Keay suggested that each year, on the morning of the day of the annual meeting, a short conference of Presidents and Vice-Presidents should be held.

H.R.H. Princess Alexander of Teck presented the Order of Mercy to seventy ladies and gentlemen.

### Sir Frederick Green wished to thank the Chairman (Lord Farquhar) for his kind words on his appointment as Treasurer. He appreciated the kindness of those remarks, as also the responsibility which he had taken upon himself.

Lord Farquhar had stated that the present year was a record one. In these dark days, when they thought they would be unable to come up to their past traditions, the result had been that the collections were £602 more than last year. The amount for the year was £19,582, and that reflected the greatest credit on the many able workers in the League and showed that the machinery of the League was simple and effective. It had enabled the £14,000 to be continued to the King's Fund, the same as last year, and what pleased the Council most was that it had been possible to fulfil the tacit promise to the voluntary hospitals that if they would exert themselves to take in as many sick and wounded as possible, the League would do its best to increase the grant. Five hundred and twenty-two pounds more had been given to them than last year. He did not propose to burden the meeting with the list of contributions from the various centres, but he would remark briefly on the districts which had collected increased amounts. He wished to specially mention the three first districts. Westminster was first with a total of £1,301, which was £1,082 more than last year. That splendid achievement was due almost wholly to Lady Kilmorey, who made a personal effort, writing or seeing all the principal people whom she thought she could usefully see or write to in her district. He regretted that she was not present to hear the applause with which the amount was received, but perhaps Mr. Harris, from the same district, would convey the information, and how much the League appreciated what her Ladyship had done. South Kensington came second with £1,150. South Kensington had nearly always held the first position, owing to the energies of Lady Chesterfield. He was not altogether sorry that South Kensington was not first, because it was more than likely it would make a special effort to recover its premier position. The third was South Paddington, presided over by that consistent worker, Lady Dimsdale, who was much assisted by Miss Enthoven. Of the other districts in which there were increases, and of which there were eighteen, he would make brief reference. Battersea showed £90 increase, Camberwell £153, owing largely to the excellent work of Miss Meadows. The City of London showed an increase of £50, and they were fortunate in the Lord Mayor having secured a grant of £100 from the Corporation, which the League had never before been able to secure. He hoped that the grant would not only be continued in the future, but that it might blossom out into a big collection. Greenwich showed £67 increase, due largely to the energy of Miss Stone; while Hampstead showed an increase of £165, for which special praise was due to the Rev. W. H. Hornby Steer and Mrs. Maclean. North Kensington showed an increase of £101, and East Marylebone £70. For the latter special thanks were due to Sir Herbert Tree for having kindly given one-third of the profits of the Sunday lectures at his theatre. West Marylebone showed a splendid increase of £431, the Chairman's district, in which he was greatly helped by Miss Olive Andrews, who contributed herself over £300, the sum collected in kinema palaces. St. George's, Hanover Square, gave an increase of £122, thanks to Colonel Hamilton's excellent results from the envelope collection and his own generous subscription. Romford and Ilford showed an increase of £94; South Hampshire, £109 (thanks to Colonel Sir William Watts); Hertford, £59; Ealing, £230 (largely due to envelope collecting and kinemas); Hornsey and Finchley, £87; Oxfordshire, £60 (due largely to the envelope system); Eye, £51; Epsom and Esher, £106; Brighton and Hove, £57, in which was included a handsome gift by Miss Pigeon of £50. Twenty-five districts took up the envelope scheme, and had between them 107,000 envelopes, and £900 had been collected by this means at a very small cost. He had been asked to mention two matters which might possibly be considered to savour of impertinence. One was to ask presidents and secretaries to be good enough to send in their collections a little earlier. *Bis dat qui cito dat* was a good quotation to bear in mind. His Serene Highness, soon after the outbreak of war, realised that the work of the League was changing, and at a meeting presided over by the Grand President it was decided to utilise the League of Mercy for the purpose of collecting. And that was what he thought they should do this year.

### Sir William Collins assured Mr. Keay that his suggestion concerning the holding of a conference on the morning of the day of the annual meeting would be considered. This was the second annual meeting which would have been held under war conditions, and again the League was able to chronicle a rising revenue. He thought enough had not been made of the fact that they were not only £600 better than last year, but that last year's figure included a legacy of £1,500, which amount also had been met this year by subscriptions. This was the best collection since 1911, when the shadow of the Insurance Act was over the voluntary hospitals, and, in the minds of some pessimists, the death-knell of the voluntary hospitals had been rung. Those who held that view had since been disabused of the notion; voluntary hospitals had shown, alike in peace and war, their usefulness. This year had been specially difficult, partly through many of our best workers being mobilised, and partly owing to the competition brought about by the many funds of charitable intent, as well as the stringency regarding money and the increased taxation. The League had kept its pledge, for forty-three out of the fifty-five hospitals which took in wounded soldiers had received a substantially increased grant compared with that made in 1913. At that time the Government had not determined on its grant-in-aid; and even so the grant did not cover more than two-thirds of the expense of maintaining the soldiers taken by the voluntary hospitals. There had been a general increase of 40 per cent. for commodities. In 1911 it was thought the State was likely to supersede the voluntary hospitals, but in 1915, after seventeen months of war, voluntary hospitals were requested to take in sailors and soldiers whom the State could not provide for. There had never been a more complete justification and vindication of the voluntary hospital system. The League was fortunate in having Lord Sandhurst to address the gathering, because no one could speak with greater authority on the voluntary hospital system. No one was better able than his Lordship to speak of the beneficent work of the King's Fund and the League of Mercy during the seventeen years the latter had existed.

After an address by Lord Sandhurst (see p. 277), the meeting closed with the usual votes of thanks.

